# DNA Ring Motif with Flexible Joints

**DOI:** 10.3390/mi11110987

**Published:** 2020-10-31

**Authors:** Shiyun Liu, Satoshi Murata, Ibuki Kawamata

**Affiliations:** 1 Department of Robotics, Graduate School of Engineering, Tohoku University, Sendai 980-8579, Japan; hikari@molbot.mech.tohoku.ac.jp (S.L.); murata@molbot.mech.tohoku.ac.jp (S.M.); 2 Natural Science Division, Faculty of Core Research, Ochanomizu University, Tokyo 112-8610, Japan

**Keywords:** DNA origami, DNA nanotechnology, molecular robotics

## Abstract

The invention of DNA origami has expanded the geometric complexity and functionality of DNA nanostructures. Using DNA origami technology, we develop a flexible multi-joint ring motif as a novel self-assembling module. The motif can connect with each other through self-complementary sequences on its segments. The flexible joints can be fixed in a straightened position as desired, thereby allowing the motif to take various shapes. We can adjust the number of flexible joints and the number of connectable segments, thereby enabling programmable self-assembly of the motif. We successfully produced the motif and evaluated several self-assembly patterns. The proposed multi-joint ring motif can provide a novel method for creating functional molecular devices.

## 1. Introduction

Structural DNA nanotechnology has been developed to assemble nanostructures using nucleic acids as a material [1]. There are two main approaches: DNA tile and DNA origami. DNA tile is an approach to self-assemble lattice-like nanostructures by using homogenous components with defined connectivity [2], which allows us to make a large 2D structure with relatively small number of base sequences, however, overall shape of the structure is difficult to be controlled. DNA origami is an approach able to self-assemble complex shapes by folding a long scaffold strand with many short staple strands [3,4,5,6]. Although it requires a large set of synthesized staples, we can assemble 2D and 3D object by DNA origami. It is the most powerful and versatile method in structural DNA nanotechnology.

In recent years, one of the focuses of the research is to give programmability to self-assembly [7,8,9,10,11]. Exploiting dexterity of DNA origami to make a programmable motif of self-assembly is typical. For example, Gerling et al. proposed a method for assembling DNA origami structures by creating complementary convexities in their shapes [12]. Tikhomirov et al. succeeded in the hierarchical assembly of DNA origami panels with different binding strengths [13]. Suzuki et al. successfully created two-dimensional lattices in the order of micrometers through the self-assembly of adhesive two-dimensional DNA origami structures floating on a lipid membrane [14]. All the techniques proposed thus far have employed motifs with determined shapes. Rigid motifs with determined shapes are suitable for making crystalline structures; however, it is difficult to modulate the morphology of self-assembled structures.

Meanwhile, aiming at realizing functional molecular devices, design of reconfigurable nanostructures by DNA origami becomes another important field of research [15,16,17,18]. For example, Kuzuya et al. proposed a plier-like nanomechical DNA origami device to detect a target molecule [19]. Douglas et al. developed a DNA origami cargo with aptamers, which is possible to be applied as a drug delivery system [20]. Funke et al. create a DNA force spectrometer to reveal the energy landscape for nucleosome association [21,22]. Upon creating complex and functional molecular devices, it is still challenging to implement a dynamic and reconfigurable nanostructure with modular programmability.

Our idea is to bring the concept of reconfigurability in the design of programmable motif for self-assembly. Here, we propose a self-assembling motif whose shape, connectivity, and flexibility are programmable. The motif, which is fabricated using the DNA origami technique, is a closed-ring structure composed of multiple joints. The joints can be either free or fixed, thereby allowing the motif to have various shapes. In addition, the joints in the motif can connect with each other through the segments. These properties enable the motif to assemble in a programmable manner with different variations.

## 2. Materials and Methods

### 2.1. Sample Preparation

The motif was prepared by mixing 10 nM scaffold strands derived from the single-stranded genome of the M13mp18 derivative (p8064, tilibit nanosystems), 50 nM of each oligonucleotide staple strand (Eurofins Genomics, Louisville, KY, USA), buffer (5 mM Tris, 1 mM EDTA, pH 8.0 at 25 °C), and salts (12.5~17.5 mM MgCl_2_). The mixture was subjected to a thermal-annealing ramp that was maintained at 85 and 65 °C for 5 and 15 min, respectively. Then it was cooled from 65 to 45 °C over the course of 20 h and finally cooled from 45 to 25 °C over the course of 20 min. For quantifying the yield of the unpurified DNA origami, motifs and their assembled structures were electrophoresed on a 1% agarose gel containing 0.5 × TBE buffer and 5 mM MgCl_2_ at 50 V for 90 min at 4 °C (Figures S1 and S2). Motif bands were stained by SYBR^®^ Gold (Thermo Fisher Scientific Invitrogen^TM^, Waltham, MA, USA) and observed using a gel imaging system (ChemiDoc^TM^ XRS Plus, Bio-Rad Laboratories, Inc., Hercules, CA, USA).

### 2.2. HS-AFM Imaging

The ring motif and its self-assembly were monitored by high speed atomic force microscopy (AFM) (Nano Live Vision, RIBM, Kyoto, Japan) with Micro Cantilevers (Olympus, Tokyo, Japan). Each measurement was performed in a liquid cell in an observation buffer (5 mM Tris, 1 mM EDTA, and 12.5 or 17.5 mM MgCl_2_). The concentration of the motif was constantly maintained at 1 nM. The substrate for AFM observation was a freshly cleaved mica (Furuuchi Chemical Corporation, Tokyo, Japan). Images were recorded with 1500 × 1125 nm^2^ scan size. The observation was conducted about 10 min after injection of the unpurified DNA origami solution. The images were analyzed using ImageJ open-source software.

## 3. Design

**Figure 1:**
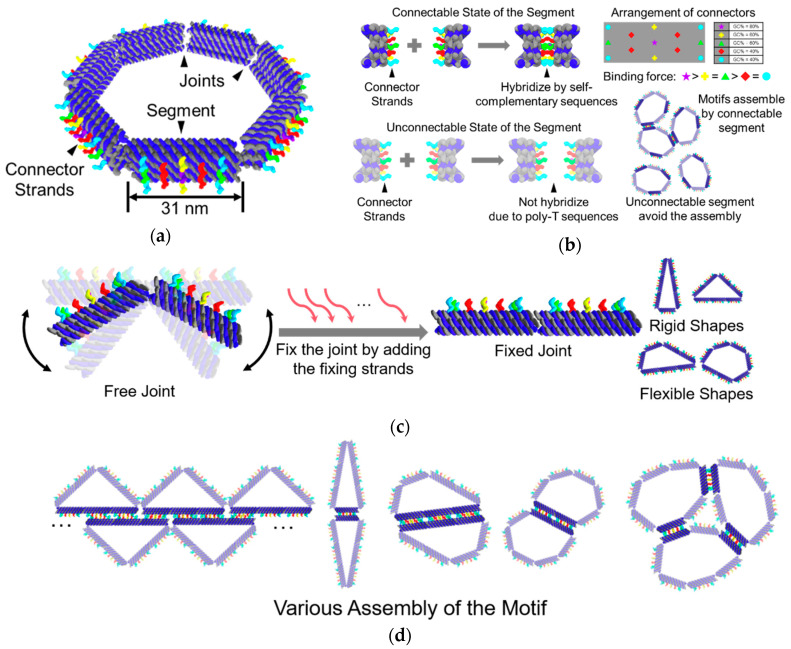
Overall design of the DNA ring motif. (**a**) Schematic of the motif in heptagonal shape; (**b**) The connector strands on the segment of the motif. The state of the segment can be selected as connectable or unconnectable. The connector strands are arranged symmetrically on the segment. The binding force are different for the connector strands at different locations. (**c**) The shape control of the motif. The motif can take different shapes by fixing the free joints at different positions; (**d**) Examples of the self-assembled structures of the motif.

We proposed the DNA ring motif as a DNA origami polygon consisting of several segments. Owing to the limitation of the length of the scaffold strand and the minimum length necessary to make a segment, we designed the DNA ring motif as an equilateral heptagon. The heptagon is the smallest equilateral polygon that can transform into two different triangles, which can provide more possibilities for the programmability of shapes. A segment in the motif is composed of 12 DNA double-helix bundles. Ten of these double-helix bundles are aligned as a 2 × 5 lattice, which creates a section of the segment. The remaining two double helices are used to link the segments to build the motif (Figure 1a).

There are 13 connector strands in each segment. The segments can be selected as either a connectable or an unconnectable state. In the connectable state, the sequences of the connector strands are self-complementary, which allows the segments to connect with another. These strands are aligned symmetrically on the outer side of the segment so that the segment can connect with each other even if the motif is flipped. To avoid dislocation among the segments, the connector strands arranged in the center of the segment are designed to have the strongest binding ability, while the connectors in the four corners are designed to have the weakest binding ability. The length of the self-complementary sequences was determined to be 10 nt by a pre-experiment (Figure S2). In the unconnectable state, the connections among the segments can be prevented by using poly T sequences instead of the self-complementary sequences (Figure 1b). 

The segments are linked by two single-stranded DNAs, which are part of the scaffold strand. Since single-stranded DNA has higher flexibility than DNA double-helix, the two single DNA strands can act as a flexible joint, which allows the motif to take different shapes. The joints of the motif can be fixed directly by adding 12 single-stranded DNAs (Figure 1c). 

The shape and connectivity of the motif can be programmed by fixing the joints and selecting the state of the segments at different positions. Utilizing the programmability in shape and connectivity of the motif, various self-assembling patterns can be achieved (Figure 1d).

## 4. Results

### 4.1. Shape Control

The shape of the ring motif can be controlled by fixing the joints at different positions. We observed the motif in the shape of heptagon and two different triangles by AFM. We chose triangles as the target of observation because triangles have a stability in shape, which are easy to observe and analyze. We measured the number of vertices, the length of the edge, the inner angles, and the perimeter of the motif to evaluate the geometric characteristics of the motif. Here, the measured length of the segment can take integral multiple values of the length of the segment in the situation where two or more segments are aligned straight.

**Figure 2:**
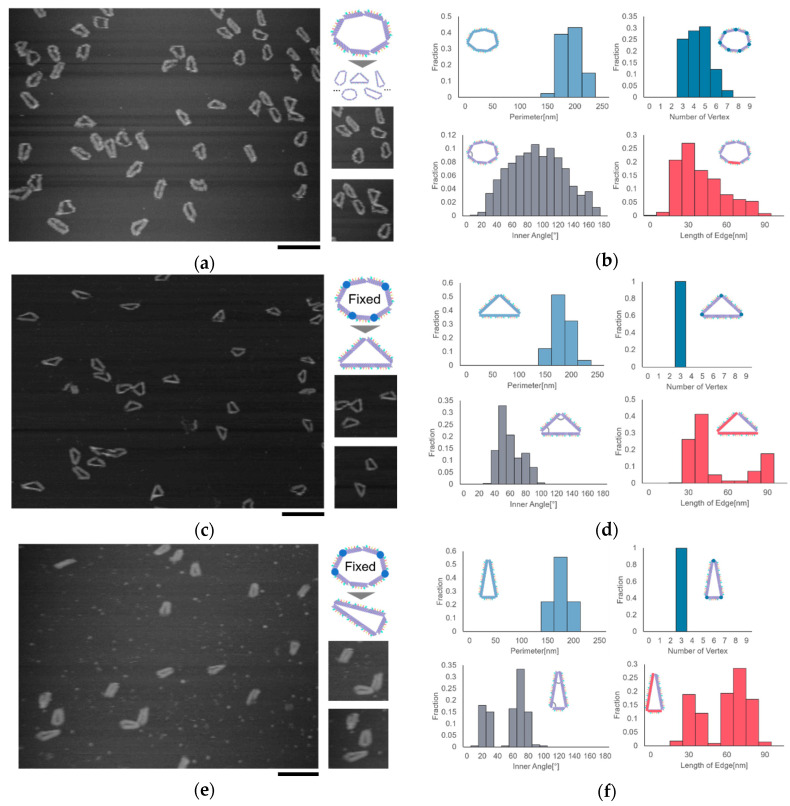
Formation and shape control of the DNA ring motif. (**a**) AFM image of the DNA ring motif in heptagonal shape. Scale bar = 200 nm; (**b**) Analysis result of the heptagonal motif; (**c**) AFM image of Triangle (2, 2, 3), scale bar = 200 nm; (**d**) Analysis result of Triangle (2, 2, 3); (**e**) AFM image of Triangle (3, 3, 1), scale bar = 200 nm; (**f**) Analysis result of Triangle (3, 3, 1).

All the joints in the heptagonal motif are set free, allowing the motif to take different shapes in addition to heptagon. The results showed that the number of vertices was distributed into different values in the range of 3–7. Moreover, the distributions of the inner angle and the length of the edge showed that the motif could take different shapes, which proved the flexibility caused by the free joints (Figure 2a,b).

The joints of the motif can be fixed by adding several single-stranded DNAs. We can determine the shape of the motif by fixing the joints at specific positions. We fabricated two types of isosceles triangles, Triangle (2, 2, 3) and Triangle (3, 3, 1), by fixing four joints of the motif in two different ways. Here, Triangle (2, 2, 3) represents an isosceles triangle with three edges consisting of 2 segments, 2 segments, and 3 segments. Two types of triangles with different shapes were observed successfully. The distribution of the inner angle and the distance between two vertices showed two peaks, indicating that the motif changed into the shape of the isosceles triangle as designed (Figure 2c–f).

### 4.2. Self-Assembled Structures

**Figure 3:**
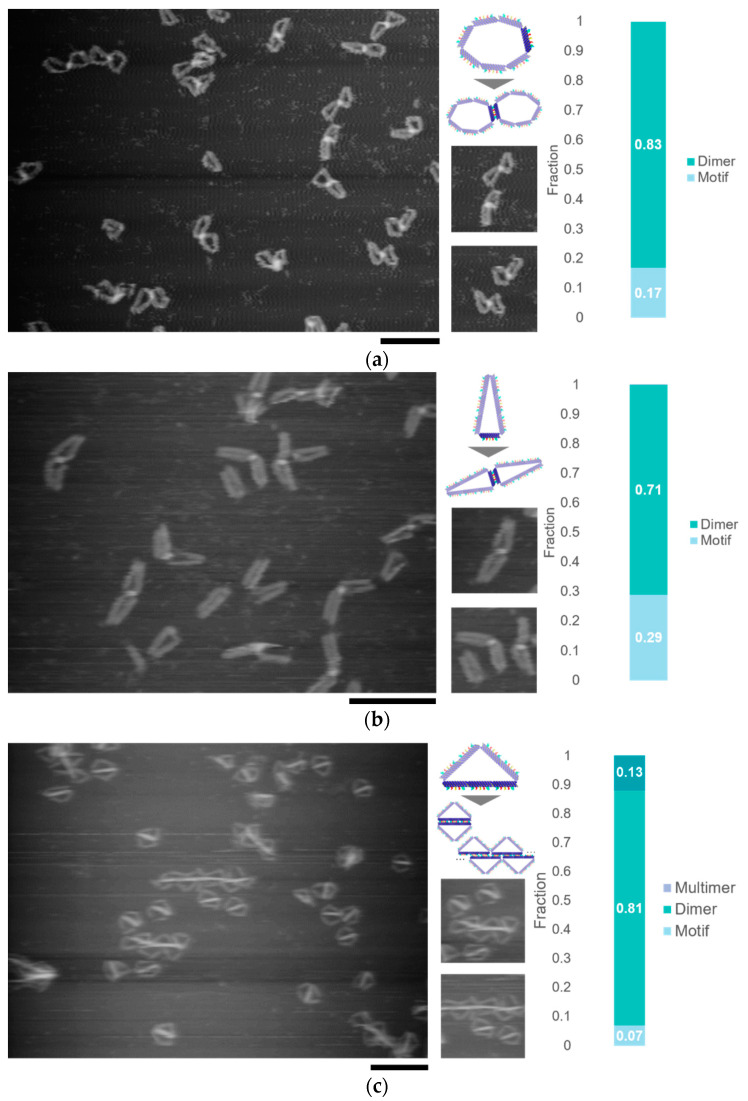
Self-assembly of the motifs with different shapes and connectivity. (**a**) AFM image of dimer formed by the DNA ring motif in flexible state; (**b**) AFM image of dimer formed by the DNA ring motif in triangle (3, 3, 1) state; (**c**) AFM image of dimer and polymer formed by the DNA ring motif in triangle (2, 2, 3) state. Scale bar = 200 nm.

The flexible DNA ring motifs in different shapes can self-assemble into various structures. By turning the segments into connectable states at different locations, it is possible to obtain different structures. We observed the self-assembly of the motif with different combinations of shapes and connectivity by AFM (Figure 3). The heptagonal motif with only one connectable segment formed dimers, and the yield of dimers was over 80%. The Triangle (3, 3, 1) motif whose shortest edge (1 segment) was in a connectable state also formed dimers, but the shape of the dimers differed from that formed by the heptagonal motif. Triangle (2, 2, 3), whose longest edge (3 segments) was in a connectable state, mainly formed dimers. Because the set of self-complementary sequences is the same, the motifs can be self-assembled into a linear multimer when the segments connect the others at a shifted position.

**Figure 4:**
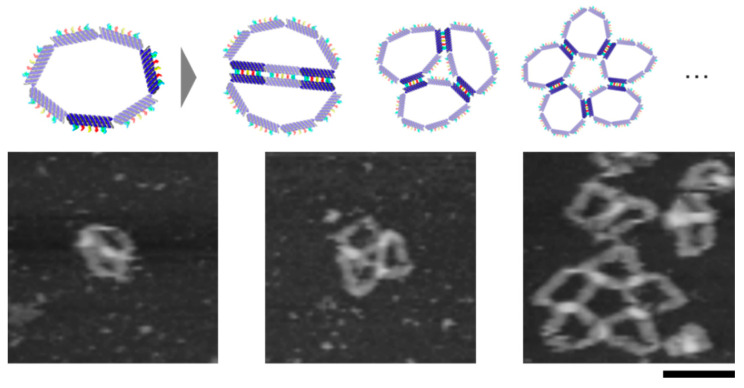
The self-assembly of the motif with two non-adjacent connectable segments. The circular multimer proved the flexibility of the motif. Scale bar = 100 nm.

We also observed the self-assembly of the motif, which has two non-adjacent connectable segments. There is one unconnectable segment between the two connectable segments. This motif can assemble circularly owing to the flexibility of the motif. AFM images showed dimers and different circular multimers (Figure 4).

## 5. Discussion

Currently, the motifs aggregated when the number of connectable segments increased. To solve this problem, the investigation of experimental conditions, the length, and the sequences of the connectors becomes necessary. Another considering reason for the aggregation is the lack of rigidity in the joint of the motif. The segment of the proposed motif consists of ten double-helix bundles arranged as a 2 × 5 lattice, while there are only two single-stranded DNAs act as the joint between the segments. The surface with a larger contact area intends to adsorb on the mica occurred during the observation of the current motif (Figure S6). Therefore, a design that can increase the rigidity of the joints is necessary. We consider making the segment by more double-helix bundles and limiting the degree of freedom of the joint to a specific direction.

As mentioned in Section 3, the segments of the DNA ring motif can be set in an unconnectable or a connectable state by using either poly T sequences or self-complementary sequences as connector. The flexibility of the joints of the motif can be adjusted by adding several single-stranded DNAs. Fixing the joints at different positions enabled us to control the shape of the motif. 

The connectivity of the motif can be programmed by adjusting the number and length of the connectors. Moreover, we can use different set of self-complementary sequences to specify the connectivity of the motif. To expand the programmability in the shape of the motif, we consider designing the motif with different numbers of segments, such as a pentagonal motif, an octagonal motif, etc. Furthermore, it is possible to assemble motifs with different shapes and connectivity into complex structures.

## 6. Conclusions

In this research, we proposed a self-assembling motif with the programmability in shape, connectivity, and flexibility. We succeeded in fabricating the motif and assembling it into different structures. The shape of the motif can be controlled by addition of fixing strands at different joints. A heptagonal motif with seven free joints and two different types of triangles with four fixed joints at different positions was successfully observed. The connectivity of the motif can be selected by setting the segments to the connectable state or the unconnectable state. Dimers with linear multimers of different shapes and circular multimers were observed.

The flexible DNA ring motif provides a novel but simple principle to form self-assembled structures in a programmable manner. In the future, this method has the potential to realize self-assembly with higher complexity on a larger scale. 

## Supplementary Materials

The following are available online at https://www.mdpi.com/2072-666X/11/11/987/s1.
