# Supplementary material for: DNA Ring Motif with Flexible Joints
Source: Micromachines (Basel). 2020 Oct 31;11(11):987. doi: 10.3390/mi11110987 (PMC7693582; doi:10.3390/mi11110987)
Supplement: Supplementary file 1 [file micromachines-11-00987-s001.pdf]

micromachines

Supplementary Information

DNA Ring Motif with Flexible Joints

Shiyun Liu [1], Satoshi Murata [1] and Ibuki Kawamata [1,2,*]

[1] Department of Robotics, Graduate School of Engineering, Tohoku University, Japan; hikari@molbot.mech.tohoku.ac.jp
[2] Natural Science Division, Faculty of Core Research, Ochanomizu University
* Correspondence: kawamata@molbot.mech.tohoku.ac.jp; Tel.: +81-022-795-6966 (F.L.)

1. Sequences of Staples

Staple	Sequence	Length
1	TCTACTAATAATCAGGTCATTGCCTGAGAGTGAGCAAAC	39
2	AAGAGAATCAACATGTTCAGCATTTTTGAGAG	32
3	GAGACTACTTTAACCTCCGGCTTAAAAGAACG	32
4	CGAGAAAACTTTTTAAAGATTCAAAGGTCTGA	32
5	GGAGACAGTCAAATCACCATAAACCAAGTACC	32
6	AACGGGTATTCAATATGATAAGAGGGTAGCTTAATGCAGAACGC	44
7	ATCTACAAAGGCTGTAGTAGCAT	23
8	AGCAATAAAGCCTGAGGCATTTT	23
9	AATAAGAAACAACGGCCTGTTATCATTCCAAG	32
10	TAATTTAGGCACAGAGCATAAAGCAATGCCGG	32
11	GGAGAAGCCTTTAAGCCTGTTTAG	24
12	TAATGTGTAGGTCAAATATATTTTTACTAGAAAATTTCAACGCAAG G	48
13	ATGACCATAATTGACCATTAGATACATTTCGATGGTCAA	39
14	TAACCTGTGTTACAAAATCGCATTCTGCGAAC	32
15	AACAACTAGATTAGAGCCGTCAATACTCGTAT	32
16	TAAATCCTTTGCCAGCTTAATTGCGGAGCACT	32
17	GCTCAACATGTTTTAAATAATATGTGAGTGAA	32
18	TAAATCAATTGCAACTAAAGGTTGATTCCCAGCAGAGGCGAATT	44
19	GAGTAGATTTAGTATCAAAAATC	23
20	AAAAAGATTAAGAACGTCAGAT	22
21	TAACAGTAGAAATTATTCATTGAAACAGTACA	32
22	TTTCAGGTTTAGGAAGCCCGAAAGATATAACA	32
23	GGAAGCAAACTCCTATAATCCTGA	24
24	GGATGGCTTAGCGAACGTTATTAACAATTCATCAAAACAGGTCAGG AT	48
25	AGCGGTCCACTAGACTGGATAGCGTCCAATACGGAATCG	39
26	TCATAAATATATTTTTGAATGCAGAGGGGGTA	32
27	GAATCAGAGGAGCTAAACAGGAGGTTTTATAA	32
28	TCAGTGAGGCCACGAATTACGAGGCCTCGTTA	32
29	CTATCATAACCCTCGTTTAAGCCAGCAGCAAA	32
30	CACGCTGAGCCAGACGACGAAGAAGTTTTGCGCTATTAGTCTTT	44
31	ATAGTAAAATGTTGCTGGTTTGC	23
32	TAAGAACTGGCTCGACCAGTAAT	23
33	ATTCTGGTACATTGAATGCGCTGCAACAGTGC	32
34	ACCAGTCACACATTATACCAGTCATTTGCAAA	32
35	CAACATTATTACACAGAACAATAT	24

36	AACGCCAAAAGCGAGTAAAAGAGTCTGGTAATATCGGTAGAAAGATTC	48
37	GCGGCCAGAATGGGCGCCAGGGTGGTTTTTCTCACCAGT	39
38	GAGACGGGATCCCTTATAAATCGCGGGGAGAG	32
39	AGAGGCAAAATACACTAAAACACTTTTGTATC	32
40	ATCGCCTGATAAACCTAATGAGTGAAAACGAA	32
41	TTGCGTTGCGCTCACTGCCAAAGGAGCGGGCG	32
42	AAGAAAGCGCGCTTTCCAGTATCGGCCAACGCAAAAGAATAGCC	44
43	GCGGTTTGCGTATTGCGGCGGGC	23
44	CTTCGCGTCCGTGTGAAACACCAG	24
45	TAAATTGTCAGTGACGAGATAGAAAGGAAGGG	32
46	TGCCCTGACGATAGCCTCCTCACAGATTAATGA	33
47	GCTGTTTCCTGTGTCAGGCGCATAG	25
48	AGCCTGGGGTGTTGTGTCGAAATCGGTGTACAGACTTGAAATTGTTATC	49
49	TTTTTTCGTCGGGTTACCTGCAGCCAGCGGTGGTGCCCC	39
50	CTGCATCACAGCTTGCTTTCGAAATCGTTAAC	32
51	TAAGTATACGGAATAGGTGTATCACGCCACCC	32
52	TCAGAGCCACCACCGCGGTTGCGGGGTTGATA	32
53	GTTGCCCTGCGGCTGGTAATCAGCAGCGAAAG	32
54	TCGTCACCCTGGGTAAAGGTGTGTGTTCAGCAGGTGAATTTCTT	44
55	GGCATCAGATGCCTCGTCGCTGG	23
56	TAGAACGTCAGCGGAATTGCGAA	23
57	TTTCACGGTGAGAAAAACAGCCTTTTGCGGGA	32
58	AACAACTAAAGTGGTGCTGGTCTGTTACACTG	32
59	CCATCCCACGCAAACGATCTAAAG	24
60	AATCCGCCGGGCCTCATTTTCAGGTAGTTAGCGTACCAGCTTACGGCT	48
61	GGTGCGGGCCTCCCGTAAAAAAAGCCGCACAGGCCTTTA	39
62	GTGATGAAATCCTCATTAAAGGTTGTGTACAT	32
63	GTGAATTACCGTCACCGACTTGAGAAGGCCGG	32
64	AAACGTCACCAATGACTTTCTCCGCATTAAAG	32
65	CTCACGGAAAAAGAGACGCTAATGCCCCCTGC	32
66	ATAAACAGTAGAAACAGCGGGCAGTTGGGCGCCAGAATGGAAAG	44
67	CGACATAAAAAAATCTTCGCTAT	23
68	GTAACGCCAGGGTACCACCACCA	23
69	CCAGCATCCACCACCGCAGTCACAGTGCCCGT	32
70	CCGCCACCAGATTTCCCAGTCACGTGCCGCCA	32
71	CCACGGGAACGGAATCTTTTCATA	24
72	TGTGAGAGATAGAAACCATCGATATAGCGTTTGCCTAACCTCACCGGA	48
73	GTTGATAATCCCAGCTTTCCGGCACCGCTTCTGCCGGAA	39
74	ACCAGGCAGCCGAACAAAGTTGGCCTCAGGAA	32
75	CCTCCCGAGCGGGAGGTTTTGAAGATCTTACC	32
76	AACGCTAACGAGCGAACAAACGGCTTAGCGAA	32
77	GATAGGTCACGTTGGTGTAGCAAAGACACCAC	32
78	AAAAGAAACGATGGGCGCATACGACAGTATCACCAGAAGGAAAC	44
79	GATCGCACTCCAGAGAAAAGCCC	23
80	ATATTTTGTTAAAAAGCCCAATA	23
81	AGAAACAGAGAGATCGAGGAATGGCAACATAT	32
82	AGAATTGAGTTATTCGCATTAAATAGGGGACG	32
83	AATAATTCGCGTCAGAATAACATA	24

84	ATTCTCCGTGGGTCTTTCCAGAGCCCTTTACAGAGTGGCCTTCCTGTA	48
85	GCTTAATTATAAAGCCTTGGACCGGTCC	28
86	ATCAATAACGTTTTTAAAATAAGGGGTTTGAATTCAAGGCCTTG	44
87	ATAGATAATCTGTCCAAGTACCGACAAAAGGTTTCAAGGCCTTG	44
88	CCATCCTCCAGTTTGTTTTGTTACCATCAAATTATGCCGGCAT	43
89	CCAACATGTATCATATTATGCCGGCAT	27
90	GGATTGACGCGCCCAGTAACAATCATCAATTATTGGCCAAT	41
91	CAAATCAGATATATGCTGATGCAAAATTTAATCGTTAAATTTATTGG CCAAT	52
92	AAGCAAGCTCGGCTGTTTACCAGTGAGAATCGTTATTGGCCAAT	44
93	CGTAACCGTGCATCTGAATTTACAGGAACGAATCAGCTTATTGGCC AAT	49
94	CATTACCCGTAATGGGCCAGCTTCCCGTCGGTTCCAATATTGG	43
95	AATCATAAAGTTAATTTCCAATATTGG	27
96	AAATTGTAAACGTTATTCCAATATTGG	27
97	CGAGCCAGTTCCAATATTGG	20
98	TATTTGCAGGTTAGAATTGGACCGGTCC	28
99	TCATTTGAGCTATTAAAGCGGAATCGGAACAATTCAAGGCCTTG	44
100	CTGAGCAACGCCTGATCATCGGGAGAAACAATTTCAAGGCCTTG	44
101	AGAAAACGATAAATTTAAATCGGCTTTTGCGTTATGCCGGCAT	43
102	GCGTAGATTTGTTTGGTATGCCGGCAT	27
103	AAGGGTGAAAAACATAATGCAATTTTAGATTATTGGCCAAT	41
104	CTTAGATTAAGACTATTAGACTTTTCATTTTGTATCATCATTATTGGC CAAT	52
105	AAATCGTCATTACCTTAATGGAAGCGTAAAACTTATTGGCCAAT	44
106	TTCAACCGTTCTAGCTAAAATTATGTAATATTGTACCTTATTGGCCA AT	49
107	ATCCTTGGAAAGGCCATAAAAATTGCCTGAGTTCCAATATTGG	43
108	GATGATGGTTTTAAAATCCAATATTGG	27
109	GAATTAGCAAAATTATTCCAATATTGG	27
110	GAATATACAGTCCAATATTGG	21
111	CTCAATCGCATGGAAATTGGACCGGTCC	28
112	GAGGCGGTGCTGAACCTCACTTGCATACTTCTTTCAAGGCCTTG	44
113	AGCCCTAACGTAAGAAGATAGAACCCTTCTGATTCAAGGCCTTG	44
114	CGAACCATTCATTCCACTTCAAAACCAGACCTTATGCCGGCAT	43
115	GCAGATTCTACCGCCATATGCCGGCAT	27
116	TGAATATATATCTGGAGAGGTCACCTTTATTATTGGCCAAT	41
117	CAAATCAACAGTTCGGTACGCCAGTTGTAGCACTGAGTAGTTATTGG CCAAT	52
118	ATCACCTTCAGTATTAAAAACGCTTCTGAAATTTATTGGCCAAT	44
119	TACGGTGTCTGGAAGTCCAGCAGAAAGCGATATCGCGTTATTGGCC AAT	49
120	CAATCAAATGCTGTATAGAGAGTATTTTTGCTTCCAATATTGG	43
121	CGGCCTTGCTGTCCATTCCAATATTGG	27
122	AAAGCGGATTGCATCTTCCAATATTGG	27
123	AAAAGGGACTCCAATATTGG	20
124	AATCAACGCAAGAACCTTGGACCGGTCC	28
125	GAAAAACCGTCACGCTGGAACCGAGAGGCGCATTCAAGGCCTTG	44
126	TGTTGTTCATGGTGGTATGGTTTAATTTCAACTTCAAGGCCTTG	44
127	TAAAGAAGAGAGGCTGGACGTTGTAACGGAATTATGCCGGCAT	43
128	ATAAGGCTGCTGGCTGTATGCCGGCAT	27
129	CATAGTAACGCTTAACTAATGCAGATTTATTATTGGCCAAT	41

130	TACAGGGCGCGTAAAGCGCGAAACGCCGGAACACTGACCATTATTGGCCAAT	52
131	GTGTAGCGGTCTATCAAATCTTGATAACAAAGTTATTGGCCAAT	44
132	TAAAAACCAAAATAGCCGTGGACAACGAACGGAAGAATTATTGGCCAAT	49
133	CCCGCCGGAGCAACAATCAGTTGAGATACATTTCCAATATTGG	43
134	AGATGAACCGCGACCTTCCAATATTGG	27
135	TACCTTATGCGATTTTTCCAATATTGG	27
136	CTTCGCGTCCGTGTGAAACACCAGAACGAGTAGTCCAATATTGG	44
137	ACAACTTTATTTCTGTTGGACCGGTCC	28
138	AGGCTTGCACGGCTACCAAACTACTAACACTGTTCAAGGCCTTG	44
139	GATAGTTGAGCCTTTACTCCAAAAAAAAGGCTTTCAAGGCCTTG	44
140	CCCACGCCCAGCTGCTTGAGGATTGGTCATATTATGCCGGCAT	43
141	TAGAAAGGTTTTGTCGTATGCCGGCAT	27
142	AGCTAACTTTTTCATAAGCATAATTCCACTTATTGGCCAAT	41
143	TTCCATTAAACGGACCCTCAGAACATGTACCGAACGCCTGTTATTGGCCAAT	52
144	GGGTAGCAAGGGAGTTGTAAATGACAACAGTTTTATTGGCCAAT	44
145	CGGGAAACCTGTCGTGATAACCGGTAATCACCCCGGGTTATTGGCCAAT	49
146	AAAGACTCACATTAACGCTCACAAAGTGTAATTCCAATATTGG	43
147	AGCCCTCAGATAGCAATCCAATATTGG	27
148	GCACGCGTGCCTGTTTTCCAATATTGG	27
149	TAATAATTTTCCAATATTGG	20
150	CCCTCAGAGAACCGCCTTGGACCGGTCC	28
151	TTAACGGGAAACATGAGCGCGTTTGTTTGCCTTTCAAGGCCTTG	44
152	TACCGTTCCCTTGATAGGTTGAGGCAGGTCAGTTCAAGGCCTTG	44
153	GATGATAAACATCCCGTCAGCAGCGGTGGTGTTATGCCGGCAT	43
154	CCTCAGAGATCAAAATTATGCCGGCAT	27
155	TATGAGCCTCAAGAGGGCGCTTCCAGCATTTATTGGCCAAT	41
156	GGATTAGCGGGGTTCACCAGTAGCAGAATCAATCATCGGCTTATTGGCCAAT	52
157	TTATTCTGGTCAGTGCCACCACCGACCGCCACTTATTGGCCAAT	44
158	TTCTTTGCTCGTCATACAGGAGTGCACCGTCAACCGCTTATTGGCCAAT	49
159	AGACTCCGGGTCACTGGAGGTGTTCGCACTCTTCCAATATTGG	43
160	CCCCTTATGCAGCACCTCCAATATTGG	27
161	AACGTGCCGGACTTGTTCCAATATTGG	27
162	GAGCCGCCGTCCAATATTGG	20
163	GAGGGTAAAACTGAACTTGGACCGGTCC	28
164	ACGTAGAATCAATAGAAAACGATTATTATTTATTCAAGGCCTTG	44
165	ATAACGGATTTTTAAGGCAATAGCTATCTTACTTCAAGGCCTTG	44
166	AAGACTCTGCTCATTACGTTGTATGGAGCCGTTATGCCGGCAT	43
167	AACCCACAAAAACAGGTATGCCGGCAT	27
168	TGGTGAAGCAAAGACACCAGTCGCGAAACTTATTGGCCAAT	41
169	GACATTCAACCGATGCACCCAGCTACAGCCATTTTTGTTTTTATTGGCCAAT	52
170	TGTCACAAAATACATAGGAGAATTTTGAGCGCTTATTGGCCAAT	44
171	ATCAAACTTAAATTTCCTTATTATCAGAGGAAACGACTTATTGGCCAAT	49
172	CCAGCGCGGATAGCTAACAATCGCCGGAATTTTCCAATATTGG	43
173	AATAGCAGCTAATTTGTCCAATATTGG	27

174	AGGCGATTAAGTTGGTTCCAATATTGG	27
175	ATAAGAGCATCCAATATTGG	20
176	GTAAAACTAGCATGTCAATCATATGTACCCCG	32
177	CTATATGTAAAGAAGGCTTATCCGGTATTCTAAGAACGCGAGGCGTT	47
178	GTATAAGCAAATATTTCAAAAACAGGAAGATTAAAGTAATGTCCTGAA	48
179	TCATTTTTACAGTAGGCAAGAAAAAGAAACCA	32
180	CCATATTTGAATATAAGACGACGACAATAAACGATGAACGGTAATC	46
181	AACGCTCATAACCAATGAGCATGTATAATATC	32
182	GCGTTATACACCGGGCACTCAGCAAGACGGTTGGGTTATATAA	43
183	CATTAAATCGTGTGATTTTTCATC	24
184	AAGAATAAACAAATTCCTTTCCTTTATCAACA	32
185	ATACCGACGTGAGCGAATAGCAAGGTAGGAAT	32
186	TCATCTTCTGACCTAATCCAATCTCGAGAAC	31
187	TTTGGGGCGCGAGCTGAAAAGGTGGCATCAAT	32
188	GGATTTAGAAGGCTGAGAAGAGTCAATAGTGAATTTATCAAAATCAT	47
189	ATACAGGCAAGGCAAATAACATCCAATAAATCAACGGATTAAGAAGAT	48
190	AAAAACATATCAAAATGATGAAACTAACAATT	32
191	AGAAATAAACCTTTTATGCTTTGAATACCAATTAGCTATATTTTCA	46
192	CCTACCATTATGACCCATTACATTAAACATCA	32
193	ATTATACATTATCATAACCTTTTCGACAAGATAATACATTTGA	43
194	ACCCTCATCCAGAAGGTTAATTTT	24
195	TATTCCTGTTCTGAATTTTTAATGTCAATTAC	32
196	AGAAACCAATATTTTAAGCGATAGCCCTTAGA	32
197	GTTTGAGTAACATTAACAAACAAGCTTCTGT	31
198	CTCAAATGCTTTAAACAGTTCAGAAAACGAGA	32
199	TTAGACAGGAAGAAAGGAATTGAGGAAGGTTATCTAAAATATCTTTA	47
200	TATTATAGTCAGAAGCAGGTCTTTACCCTGACCCTGAAAGAACATCGC	48
201	TTTTAATTTTTTGACGCATTAAAAACAGAGGT	32
202	GGATTATTCCAACAGATACGTGGCACAGACAATTCATTGAATCCCC	46
203	TACCTACACGAGCTTCAAGATAAAATACCGAA	32
204	GCCATTGAAACTATTGAAAAAGAAGTGTCCGATTAAAGGGATT	43
205	ATTGCTCCTAATAACATCAAATAT	24
206	AAGAACTCCAACAGGAACACCGCCGAACTGAT	32
207	TTGATTAGTTTTGATATCAGTTGGCAAACCCT	32
208	CACGCAAATTAACCGAATCCTGATCTAAAGC	31
209	CTTCACCGCCTGGCCCCCTGTTTGCAGTTTGG	32
210	GCGATTATACCCTATGGTTGCTTTGACGAGCACGTATAACGTGCTTT	47
211	TTTAATCATTGTGAATCCCAGCAGGCGAAAATTGAGAGAGTTGCAGCA	48
212	AAATCTACATTACCCAAACAAGAGCAAAGGGC	32
213	CTGCTCATGGCTTGAGTCCGAAATCGGCAAACAACAGCTGATTGCC	46
214	GGATATTCGTTAATAATCCAACGTTCCACTAT	32
215	ACCTTCAAGAGGACCTAGGGCACGGAGACATCTTTGACCCCCA	43
216	GGAATACCATCATAAGGCGCGTAA	24
217	ACTTTGAATCAAGAGTCGTGGCGAGGGTTGAG	32
218	GACGGTCAACATTCAATGCGCCGCCCACCACA	32

219	GCTCCATGTTACTTAAAAGTACAGCTGGCAA	31
220	TGTCACTGCGCGCCTGTGCACTCTGTGGTGCT	32
221	TTAGTACCGCCGTAAAATACGTAATGCCACTACGAAGGCACCAACC T	47
222	CCGGGGGTTTCTGCCACGTTTTCACGGTCATACCAAAAGGCGCCGA CA	48
223	TACCGAGCTTTGCTAAATGACAACGGTCGCTG	32
224	TCAGCGGATTGAAAATATTGTATCGGTTTATGACGATCCAGCGCAG	46
225	TATGGGATTCGAATTCATATATTCAACCATCG	32
226	TCTTTCCCACAGACACAGCATTCAGAACCCGTACTCAGGAGGT	43
227	ACAACATACACCAGTAAGAGGCTT	24
228	TAGCATTCAGACGTTAAAAGGCCGTTGATACC	32
229	AGTTTCGTCGAGCCGGGAGGAAGTTGAGGACT	32
230	GCCCAATAGGAACCCCGCCACCCCGGAACGA	31
231	ATGCTGATTGCCGTTCCGGCAAACGCGGTCCG	32
232	AGCCAGCAAAATTTGCTCAGTACCAGGCGGATAAGTGCCGTCGAGA G	47
233	CACATCCTCATAACGGCAGCCTCCGGCCAGAGACGATTGGCAGTAA GC	48
234	AAGAATGCAGCCGCCAGTCATACAAATAAGTT	32
235	CCTCAGAGTGACAGGATTCACAAACAAATAAGGGTAAAGTTAAAC G	46
236	TCCCTCAGCAACGGCAGTACTGGTTGGCTTTT	32
237	CACCGGATCATAGCCTATTTCCATTAGCCCATTTGGGAATTAG	43
238	CAGCGGGGAGACTGTAAAGTATTA	24
239	ATTTTCGGACCAGAGCCTTGAGTATCTGAATT	32
240	TTAGCGTCTCATTGCAAAGGATTAAGAGGCTG	32
241	GTAATCAGTAGCGACACCATTACGGAACCTA	31
242	TTCAGGCTGCGCAACTGTTGGGAAGGGCGATC	32
243	AGTTGCTATTTTTGAGGGAGGGAAGGTAAATATTGACGGAAATTATT	47
244	AGGGGGATGTGCTGCATACGCCAGCTGGCGAACGAAGCCCATACCC AA	48
245	GGCCAGTGCAAAGTCAAAGAACTGGTTAGCAA	32
246	TAATATCAATGAAATAAAAAGTAAGCAGATAAAGCGCCATTCGCCA	46
247	ACCCTGAACCAAGCTTCGCAGTATGCATGATT	32
248	GAAGCGCAAATGAAGGAATAAATCCTGACCTTAAATCAAGATT	43
249	GTACAGCGCAAATAAGAAATTCAT	24
250	AACGTCAAATTAGACGCATAAAGGACGCAATA	32
251	TCCCAATCCCATGTTTAAAAGGGCATGGTTTA	32
252	CCAGTTACAAAATAAACAATTTTGTTTATTT	31

2. Connector Strands

2.1. The Concentration of Connector Strands

To study the optimal connector concentration for the self-assembly of the DNA ring motif, we carried out agarose electrophoresis. The concentration of staple, connector and scaffold is in a ratio of 5:(5,4,3,2,1.5,1):1. The results indicated that staple : connector : scaffold = 5:5:1 leads to the highest dimer yield.(Figure S1)

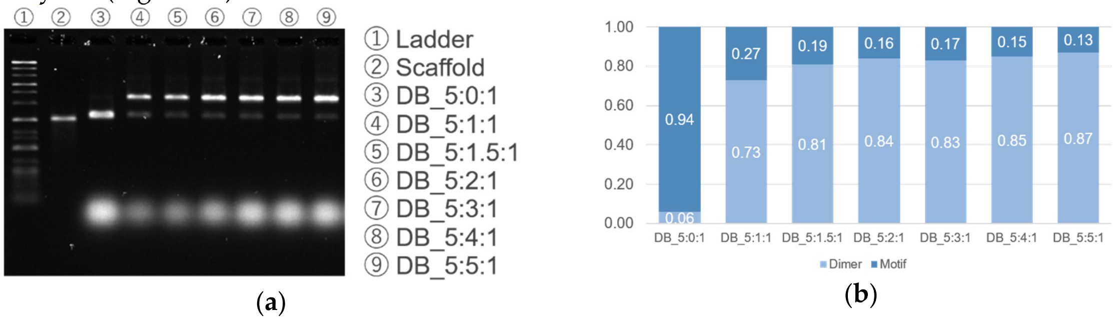

(**a**) (**b**)

Figure S1. Investigation of the concentration of the connector strands. (**a**) Agarose electrophoresis result; (**b**) Analysis result of agarose electrophoresis.

2.2. The Length of Connector Strands

We carried out agarose electrophoresis of the motif with one connectable segment to study the optimal length of the connector strands. The lengths of the self-complementary sequences were distributed from 4 nt to 20 nt in a step of 2 nt. When the length of the connector strands was 10 nt, the yield of dimers was the best. When the length of connector strands was above 12 nt, the connector strands began to hybridize with themselves and forming hairpin structures, due to their self-complementary sequences. This result indicated that 10 nt was the optimal length of the connector strands. (Figure S2)

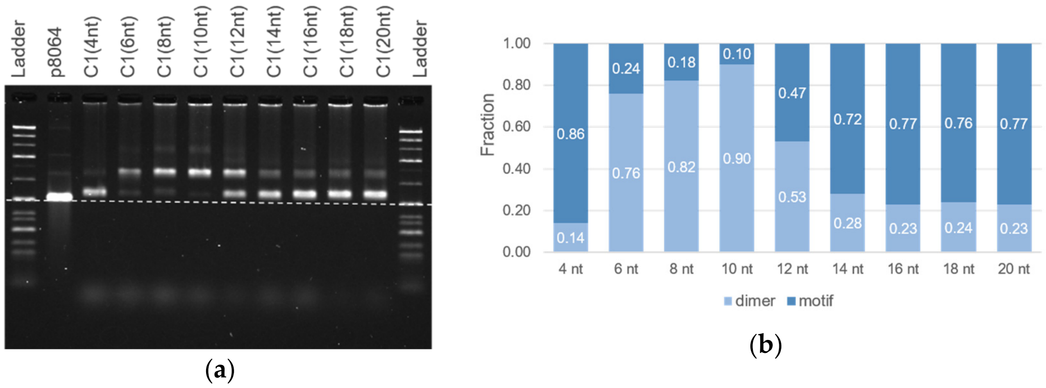

(**a**) (**b**)

Figure S2. Investigation of the length of the connector strands. (**a**) Agarose electrophoresis result; (**b**) Analysis result of agarose electrophoresis.

2.3. The sequences of connector strands

In the experiment of observing the motif and its self-assembled structures, Poly T_4 nt is used for the unconnectable segments. Self-complementary_10 nt is used for the connectable segments.

**Name**	**Sequence**	**GC%**
Poly T_4 nt	TTTT	0%
Self-complementary_4 nt	GCGC	100%
	CGCG	100%
	CATG	50%
	AGCT	50%
	TATA	0%

Self-complementary_6 nt	CGGCCG	100%
	AGCGCT	67%
	TCGCGA	67%
	TACGTA	33%
	AGTACT	33%
Self-complementary_8 nt	GGACGTCC	75%
	CATGCATG	50%
	GTACGTAC	50%
	AGATATCT	25%
	TTCATGAA	25%
Self-complementary_10 nt	GGACCGGTCC	80%
	CAAGGCCTTG	60%
	ATGCCGGCAT	60%
	ATTGGCCAAT	40%
	CCAATATTGG	40%
Self-complementary_12 nt	GCTCCGCGGAGC	83%
	GACGTCGACGTC	67%
	AGCCTGCAGGCT	67%
	ATCGATATCGAT	33%
	GTCAGCGCTGAC	33%
Self-complementary_14 nt	GCCTGGCGCCAGGC	86%
	GACGTGCGCACGTC	71%
	CTGGCCATGGCCAG	71%
	ATGCAGGCCTGCAT	57%
	GCTTGACGTCAAGC	57%
Self-complementary_16 nt	GCCGAGGCGCCTCGGC	87.5%
	GACGTCCGCGGACGTC	75%
	ACCGTGGCGCCACGGT	75%
	TGCAGATCGATCTGCA	62.5%
	GTAGCAGGCCTGCTAC	62.5%
Self-complementary_18 nt	GCCGACGGCGCCGTCGGC	89%
	AGGCGTGCCGGCACGCCT	78%
	GCCAGGTCCGGACCTGGC	78%
	GAAGCTCGGCCGAGCTTC	67%
	TGGCGTGCATGCACGCCA	67%
Self-complementary_20 nt	GCCCAGCGGCGCCGCTGGGC	90%
	AGGCGTCCGGCCGGACGCCT	80%
	CGGGACGTCCGGACGTCCCG	80%
	CTGGTACCGCGCGGTACCAG	70%
	AGCCAGGCTGCAGCCTGGCT	70%

3. Shape Control

3.1. Heptagonal Motif

Heptagonal motif has no connectable segment, and all the seven joints are flexible. The AFM images showed that the heptagonal motif formed successfully and took different shapes due to its flexibility.

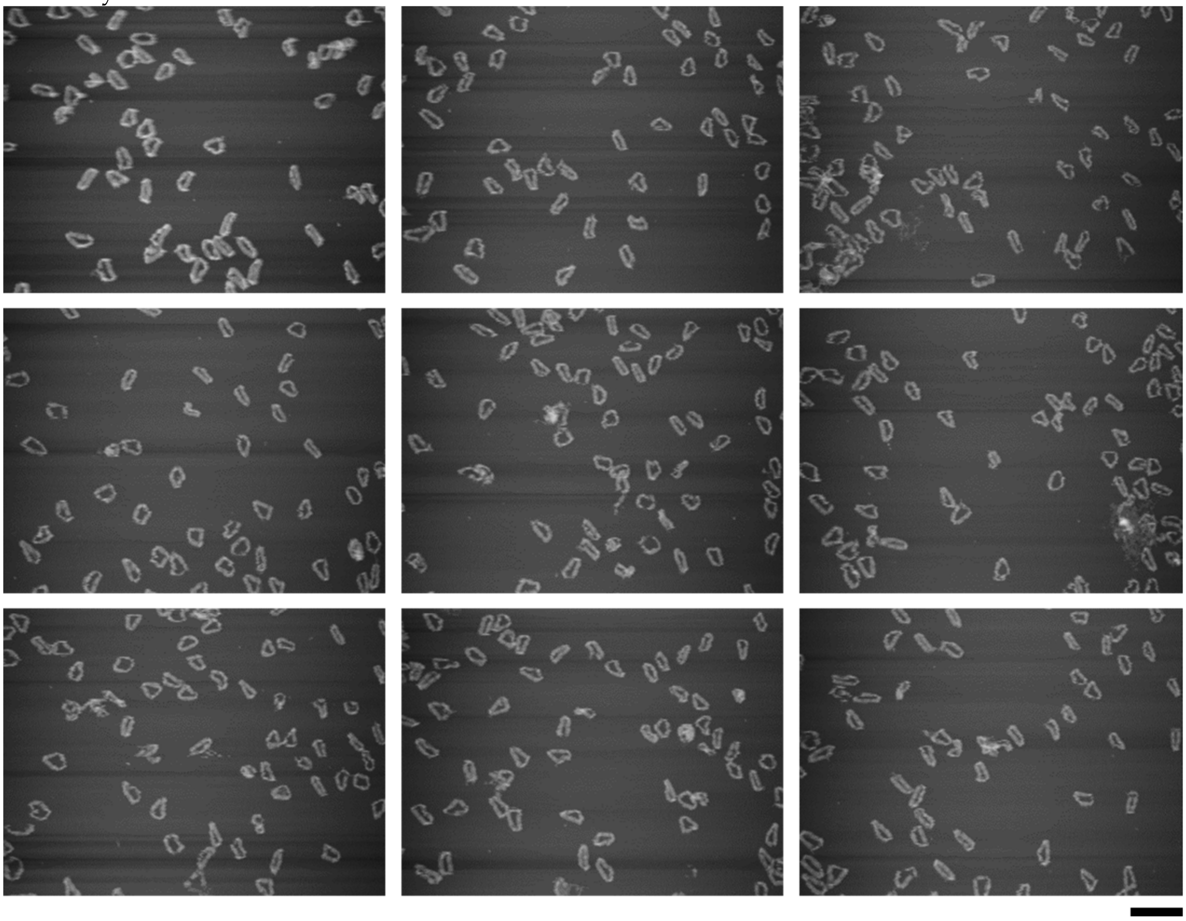

Figure S3. AFM images of the heptagonal motif. Scale bar = 200 nm.

3.2. Triangle (2,2,3)

Triangle (2,2,3) is the triangle motif whose edges are in the length of 2 segments, 2 segments, 3 segments, respectively. All the segments are unconnectable. Four joints are fixed into the straight state. The AFM images showed that Triangle (2,2,3) formed successfully.

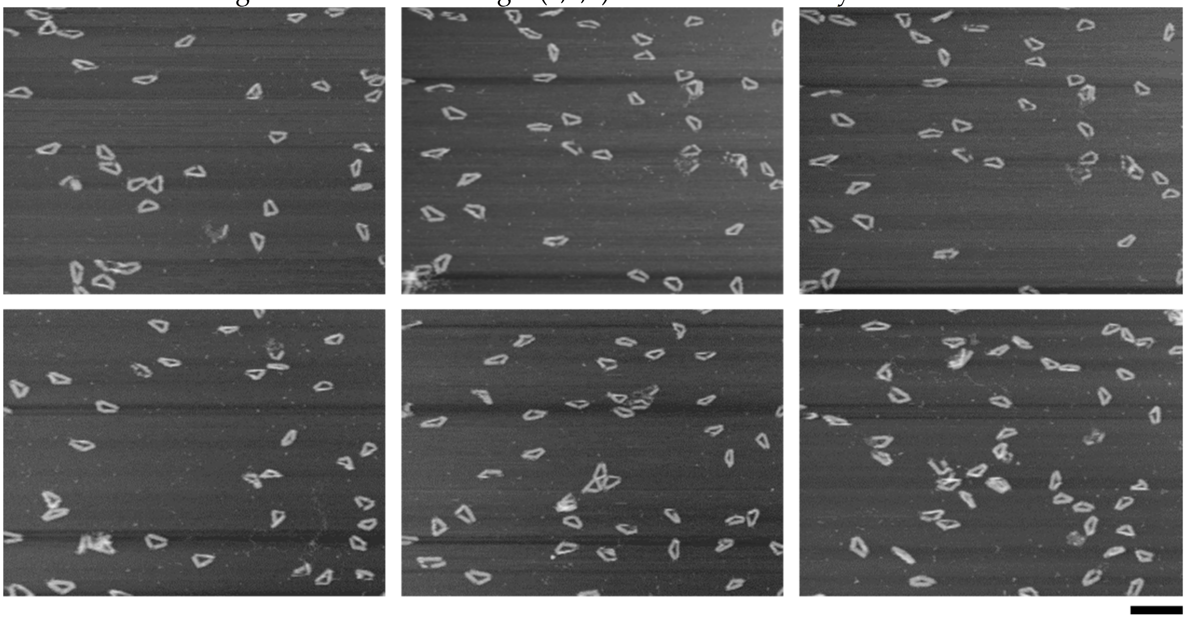

Figure S4. AFM images of Triangle (2,2,3). Scale bar = 200 nm.

3.3. Triangle (3,3,1)

Triangle (3,3,1) is another kind of triangle motif whose edges are in the length of 3 segments, 3 segments, 1 segment, respectively. All the segments are unconnectable. Four joints are fixed into the straight state. The AFM images showed that Triangle (3,3,1) formed successfully.

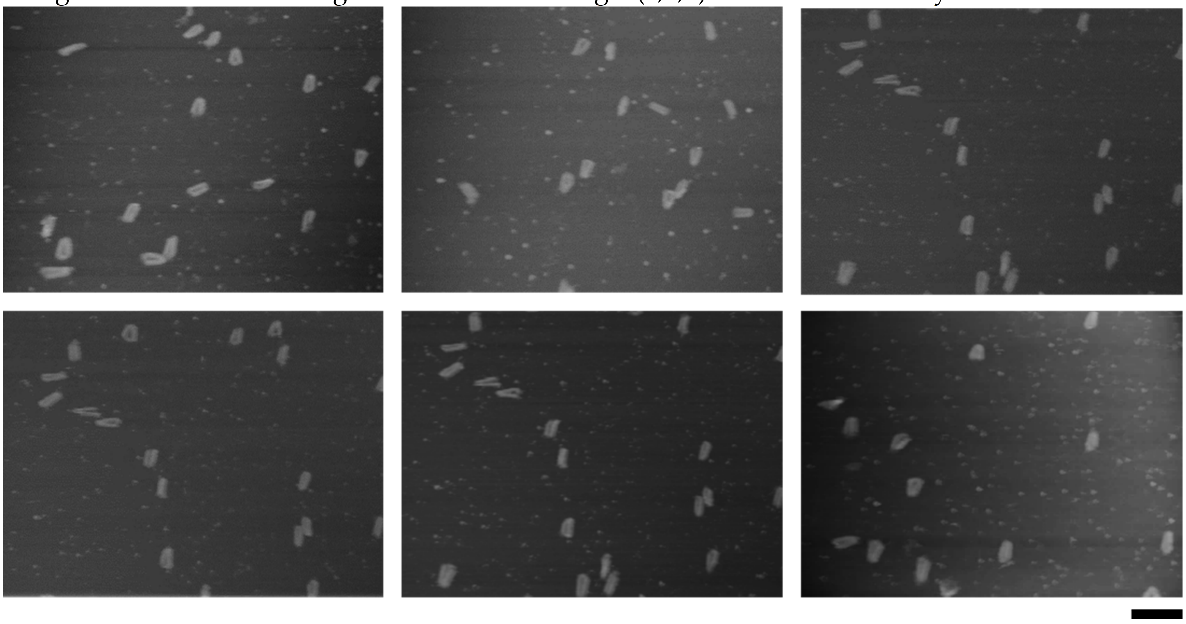

Figure S5. AFM images of Triangle (3,3,1). Scale bar = 200 nm.

4. Self-assembled Structures

4.1. Self-assembly of the heptagonal motif

We observed the self-assembly of the heptagonal motif which has one connectable segment and six unconnectable segments. The AFM images showed that the motif formed dimers as expected.

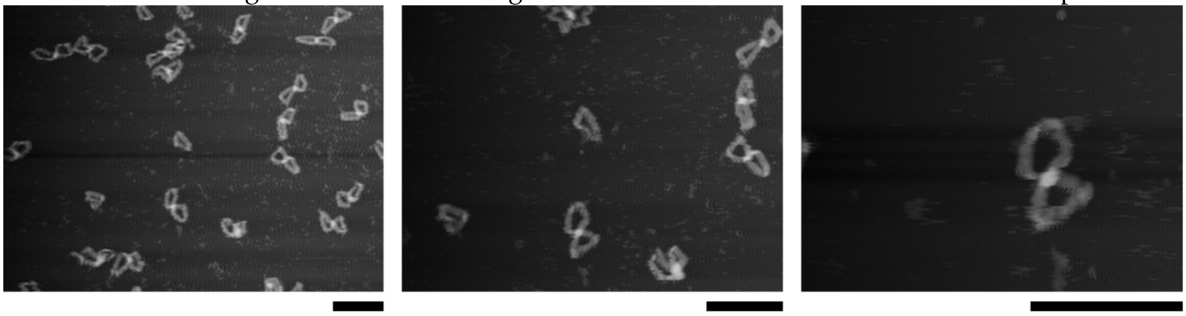

(**a**)

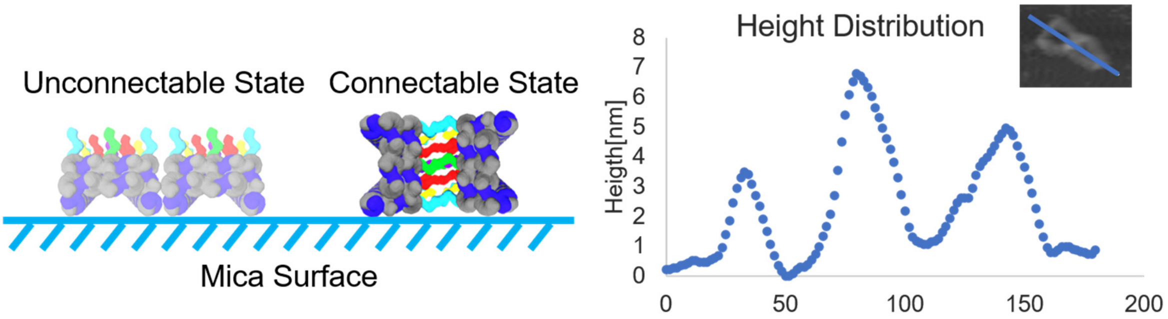

(**b**)

Figure S6. (**a**) AFM images of the dimer formation of the heptagonal motif. Scale bar = 200 nm; (**b**) Height distribution of the dimer formed by the heptagonal motif.

4.2. Self-assembly of Triangle (3,3,1)

We observed the self-assembly of Triangle (3,3,1). The segment acting as bottom edge is connectable while the rest six segment are unconnectable. The AFM images showed that the motif formed rhombus-shaped dimers as expected.

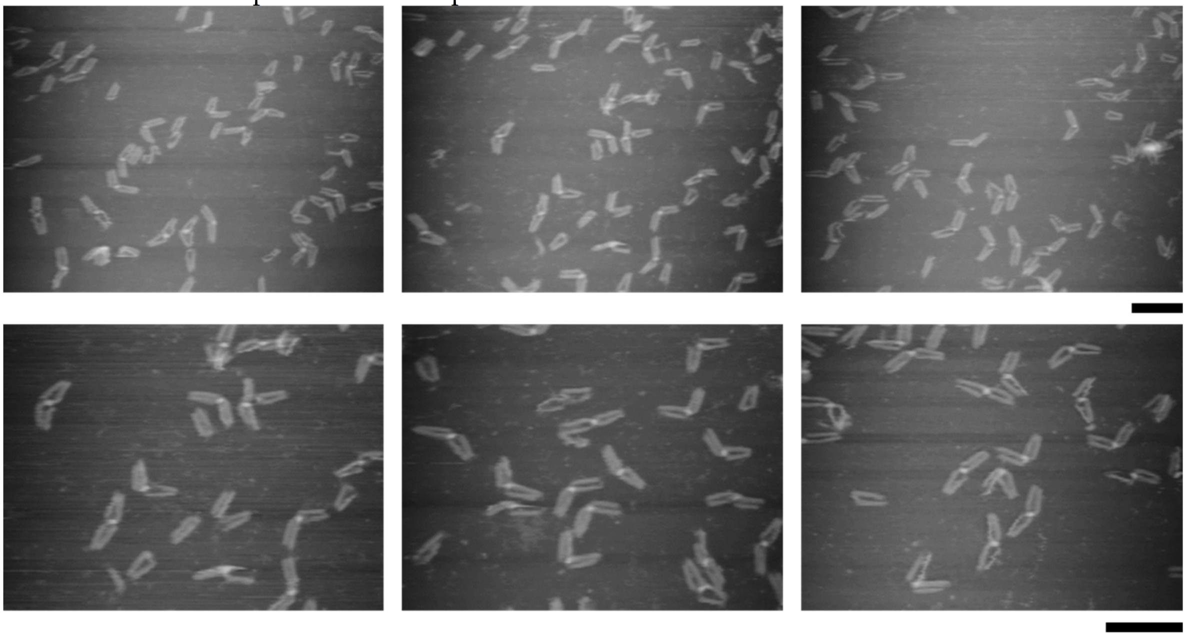

Figure S7. AFM images of the dimer formation of Triangle (3,3,1). Scale bar = 200 nm.

4.3. Self-assembly of Triangle (2,2,3)

We observed the self-assembly of Triangle (2,2,3). The three segments acting as bottom edge are connectable while the rest four segment are unconnectable. The AFM images showed that the motif formed not only rhombus-shaped dimers but also linear-aligned polymers since there is only one kind of the self-complementary sequences for the connector strands.

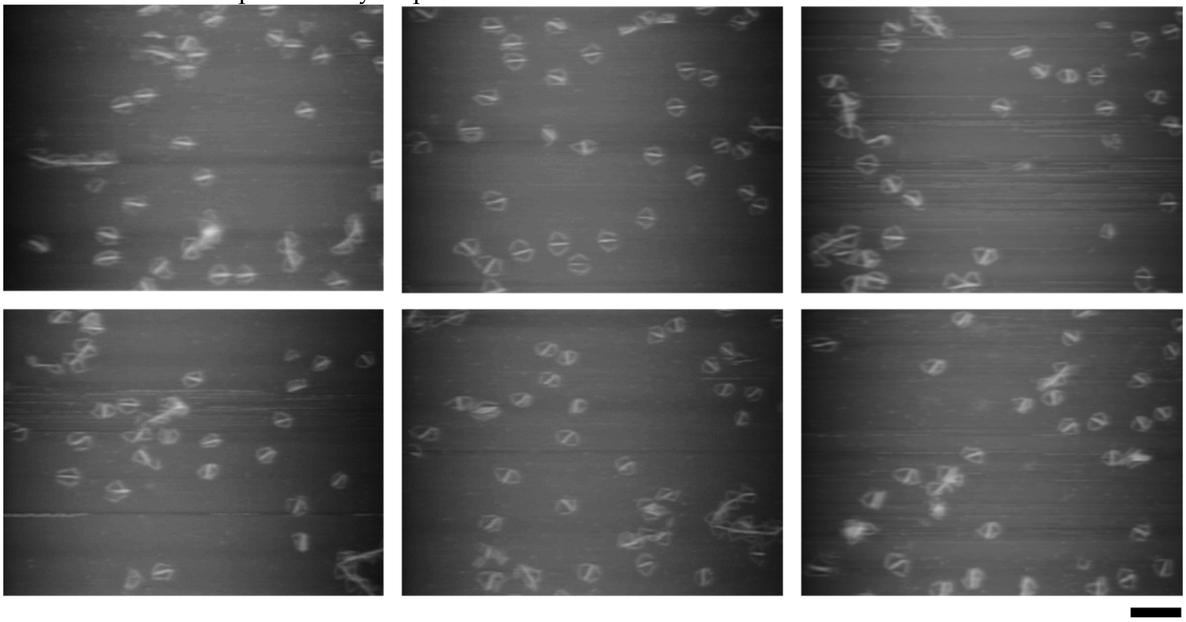

Figure S8 AFM images of the dimer and polymer formation of Triangle (2,2,3). Scale bar = 200 nm.

4.4. Circular Multimer

We also used transmission electron microscope (JEOL JEM-2100F, JAPAN) to observe the self-assembly of the motif, which has two non-adjacent connectable segments. There is one unconnectable This motif can form circular assembly due to the flexibility of the motif. TEM images showed that the motif formed circular multimers with different number of motifs.

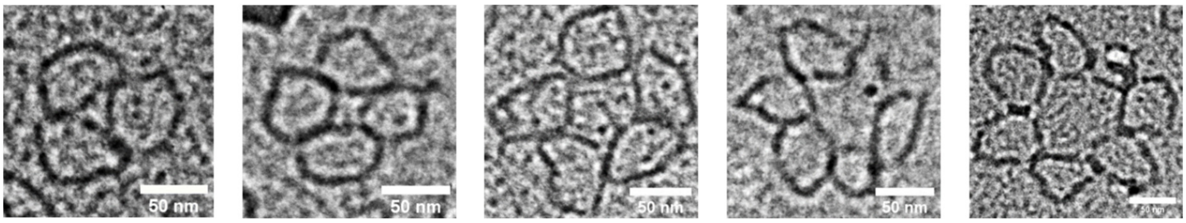

Figure S9 TEM images of the circular multimer formation. Scale bar = 50 nm.

© 2020 by the authors. Licensee MDPI, Basel, Switzerland. This article is an open access article distributed under the terms and conditions of the Creative Commons Attribution (CC BY) license (http://creativecommons.org/licenses/by/4.0/).
